# Wnt pathway in bone: knowledge structure and hot spots from 1993 to 2022

**DOI:** 10.3389/fphys.2023.1279423

**Published:** 2023-11-16

**Authors:** Tun Liu, Jiaxin Zhao, Xinyi Zhang, Yulin Wang, Wei Wang, Jidong Song

**Affiliations:** The Second Affiliated Hospital, Xi’an Jiaotong University, Xi’an, Shaanxi, China

**Keywords:** Wnt pathway, bone, osteoporosis, sclerostin antibody, multiple myeloma, DKK1, bibliometric analysis

## Abstract

**Background:** The role of the Wnt pathway in bone and its targets in skeletal disease has garnered interest, but the field lacks a systematic analysis of research. This paper presents a bibliometric study of publications related to the Wnt signaling pathway in bone to describe the current state of study and predict future outlooks.

**Methods:** All relevant articles and reviews from 1993 to 2022 were collected from the Web of Science Core Collection (WoSCC). Bibliometric analysis and visualization were performed using CiteSpace 6.1 R3, VOSviewer 1.6.15, and the Online Analysis Platform of Literature Metrology (http://bibliometric.com/).

**Results:** A total of 7,184 papers were retrieved, authored by 28,443 researchers from 89 countries/regions and published in 261 academic journals. The annual publication numbers peaked in 2021. China and United States are the leading countries, with the University of California and Harvard University as the most active institutions. Wang, Yang is the most prolific author. Bone has the most published research, while Proceedings of the National Academy of Sciences of the United States is the most cited journal on average. The main keywords include expression, Wnt, osteoporosis, bone, and osteogenic differentiation. Current and developing research hotspots focus on bone mass, sclerostin antibody, multiple myeloma, and cartilage development.

**Conclusion:** This paper provides new insights for researchers to delve into the mechanisms of Wnt and bone related diseases and translate into clinical studies. It reveals the development and future research trends in Wnt and skeletal-related studies.

## 1 Introduction

Bone is a dynamic organ that undergoes constant regeneration and continuous renewal. The systemic bone mass and skeletal health is achieved through the finely regulated process of bone remodeling, which involves a spatiotemporal balance between bone formation by osteoblasts and bone resorption by osteoclasts. This process is strictly regulated by multiple systemic hormonal and growth factor signals (Siddiqui and Partridge, 2016; Kim and Koh, 2019). Any changes in local or systemic factors modulating the bone remodeling process can result in abnormal bone homeostasis and contribute to the disruption of the delicate balance, resulting in bone-related diseases such as osteoporosis and delayed or non-healing fractures (Negishi-Koga and Takayanagi, 2012). Clinically, despite the availability of medications targeting the regulation of bone remodeling processes, challenges persist in terms of efficacy and implementation of appropriate treatments (Noh et al., 2020). Therefore, elucidating the mechanisms affecting the crosstalk between osteoblasts and osteoclasts during bone metabolism is essential for the safe management of skeletal disorders and the development of effective therapies targeting novel regimes.

The Wnt signaling is a crucial cell fate determining molecules that regulates cell proliferation, migration and polarity. The relationship between the Wnt pathway and bone was first discovered when mutations in the low-density lipoprotein-5 (LRP5) molecule, which is associated with the Wnt cascade, affected bone mass (Gong et al., 2001). Recent years, studies on Wnt pathway-mediated osteogenic differentiation of mesenchymal stem cells (BMSCs), osteogenesis, and bone repair and regeneration have received extensive attention (Nusse and Clevers, 2017; Houschyar et al., 2018). *In vivo*, there are two pathways of Wnt signaling, canonical and non-canonical pathways. The canonical pathway consists of Wnt ligands binding frizzled (FZD) receptors complexed with LRP5/6 co-receptors and activating β-catenin, allowing it to accumulate and translocate to the nucleus, inducing the expression of target genes (Baron and Kneissel, 2013). The Wnt classical pathway inhibitors, such as sclerostin antibodies and dickkopf 1 (DKK1), have shown clinical potential for the treatment of osteoporosis (Delgado-Calle et al., 2017; Colditz et al., 2018). Moreover, Wnt6, Wnt10a, and Wnt10b promote MSC osteogenic differentiation and inhibit adipogenic differentiation through the canonical Wnt/β-catenin pathway (Visweswaran et al., 2015; Maeda et al., 2019). The canonical Wnt/β-catenin pathway can also modulate bone resorption indirectly through the osteoprotegerin (OPG)/receptor activator of nuclear factor kappa beta (RANK)/receptor activator of nuclear factor kappa beta ligand (RANKL) axis (Yang et al., 2020). The non-canonical Wnt signaling pathway is independent of β-catenin and is activated by the binding of Wnt ligands to FZD receptors or FZD/Ror5/1 complexes. It has been reported that Wnt5a and Wnt7b may engage the non-canonical pathway and affect osteoblast differentiation, whereas Wnt16, Wnt5a, and Wnt4 may promote or inhibit bone resorption after activating the non-canonical pathway (Chang et al., 2007; Maeda et al., 2012; Movérare-Skrtic et al., 2014; Karner and Long, 2017). In addition, deletion of the Secreted Frizzled-related protein-4 (sFRP4) triggers both canonical and non-canonical Wnt signaling pathways and also modulates crosstalk between Wnt and BMP signaling (Kiper et al., 2016). These evidence indicates that non-canonical Wnt signaling pathways have an equally significant role in bone mass homeostasis. These imply a crucial role for the regulation of β-catenin-dependent or -independent Wnt signaling in the maintenance of bone homeostasis and offer a novel therapeutic target for osteoporosis and other skeletal disorders. Although the literature on Wnt signaling pathway-related osteogenic differentiation is gradually accumulating, a comprehensive and systematic analysis of the evolution and trends of research in this field is still lacking.

Bibliometrics assesses information on countries, institutions, journals, authors, and keywords that are relevant to the field by synthesizing data from published papers on a specific topic, and quantitatively and qualitatively analyzes it. Researchers use bibliometric tools such as CiteSpace, VoSviewer, and the Online Analysis Platform of Literature Metrology (http://bibliometric.com/) to visually analyze literature output and trends, and facilitate scholars to stay up-to-date with future research trends (Chen et al., 2022). To our knowledge, no published reviews have analyzed studies on the relationship between bone and the Wnt signaling pathway, although some researchers have used bibliometrics to count manuscripts on the Wnt signaling pathway (Xu et al., 2021) and its role in liver disease (Jiang et al., 2019), Therefore, this report presents a bibliometric study of publications related to the Wnt signaling pathway in bone over the last 3 decades (1993–2022) in order to describe the research frontiers and predict the study outlook, and to give inspiration and strategies to scholars.

## 2 Methods

### 2.1 Search strategy and data extraction

**FIGURE 1:**
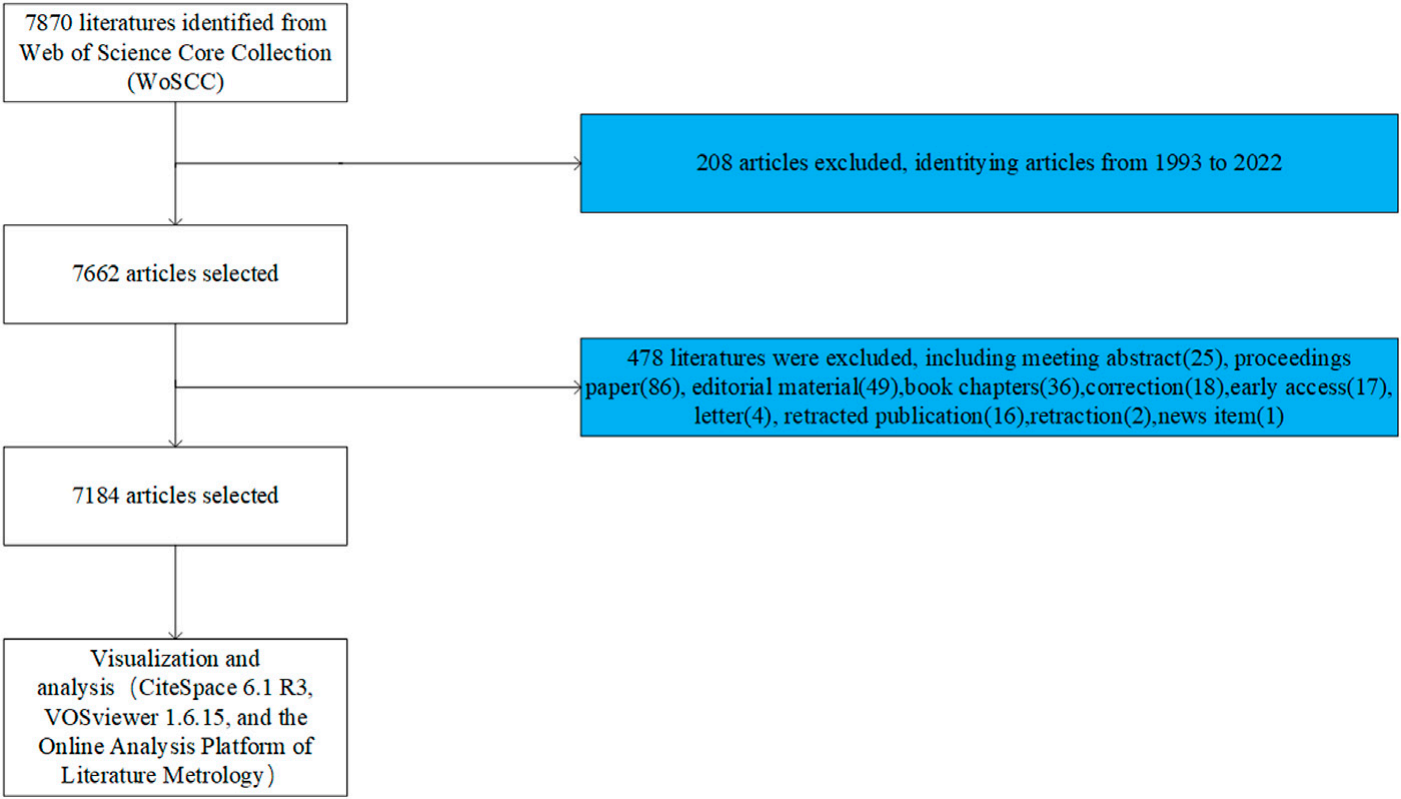
Flowchart of the screening procedure.

This article did a literature search on the Web of Science (WOS) core database on 23 June 2023. The search formula was TS = [Wnt (Topic) or Wnt signaling pathway (Topic)] and [bone (Topic)]. The year of publication of the article was set from 1993.01.01 to 2022.12.31 without any language limitation. And original articles and reviews were selected as the publication categories. A total of 7,184 results were found, which were then screened and checked by 2 reviewers to obtain literature data, including title, abstract, keywords, names of authors, nationality, affiliation, journal, and number of citations (Figure 1). We have saved the all retrieved documents in full record and cited reference formats in Excel and uploaded them as Supplementary Material.

### 2.2 Bibliometric analysis and visualization

The resulting bibliographic data were converted to. txt format and imported into CiteSpace 6.1. R3, 64-bit (Drexel University, Philadelphia, PA, United States), VOSviewer 1.6.15 (Leiden University, Leiden, Netherlands) and the Online Analysis Platform of Literature Metrology (http://bibliometric.com/) with impact factors and category quartiles from the 2021 Journal Citation Report to implement further bibliometric analyses. Citespace software was utilized for co-citation clustering analysis of references and keywords, cluster analysis of timeline views, and burst citation analysis (Chen, 2006). Vosviewer software was used to accomplish co-occurrence analysis of keywords and to examine research hotspots. Where the size and color of the nodes represent the number and category of clusters; the thickness of the line indicates the strength of the relationship between items (van Eck and Waltman, 2010). The Online Analysis Platform of Literature Metrology (http://bibliometric.com/) was engaged to visualize and analyze the contribution and collaboration of countries, institutions, and authors (Aria and Cuccurullo, 2017).

## 3 Results

### 3.1 Growth trend of publications

**FIGURE 2:**
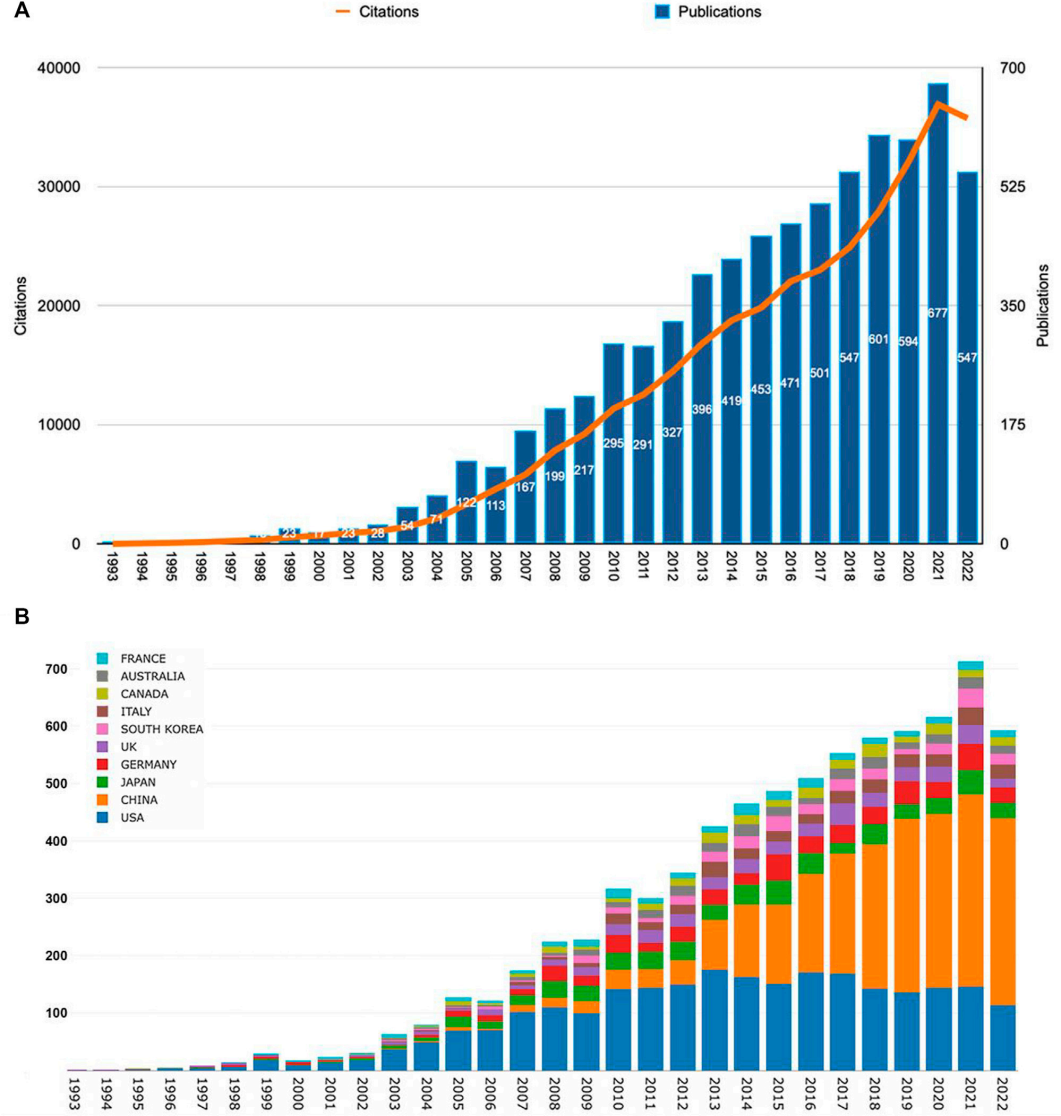
The Wnt and Bone-related articles globally. **(A)** Trends in the number of Wnt and Bone-related articles. **(B)** The Top10 countries/regions on meniscus extrusion research from 1993 to 2022.

According to our search pattern, 7,184 original publications were produced worldwide from 1993 to 2022. As shown in Figure 2A, the number of Wnt and Bone-related articles has increased each year, starting from 4 in 1993 to 547 in 2022 and peaking at 677 in 2021. The majority of articles were published after 2009. During 2018–2022, Wnt in Bone research reached a peak with 2,966 articles published in 5 years, accounting for 41% of the total.

### 3.2 Analysis of countries/regions and institutions

**FIGURE 3:**
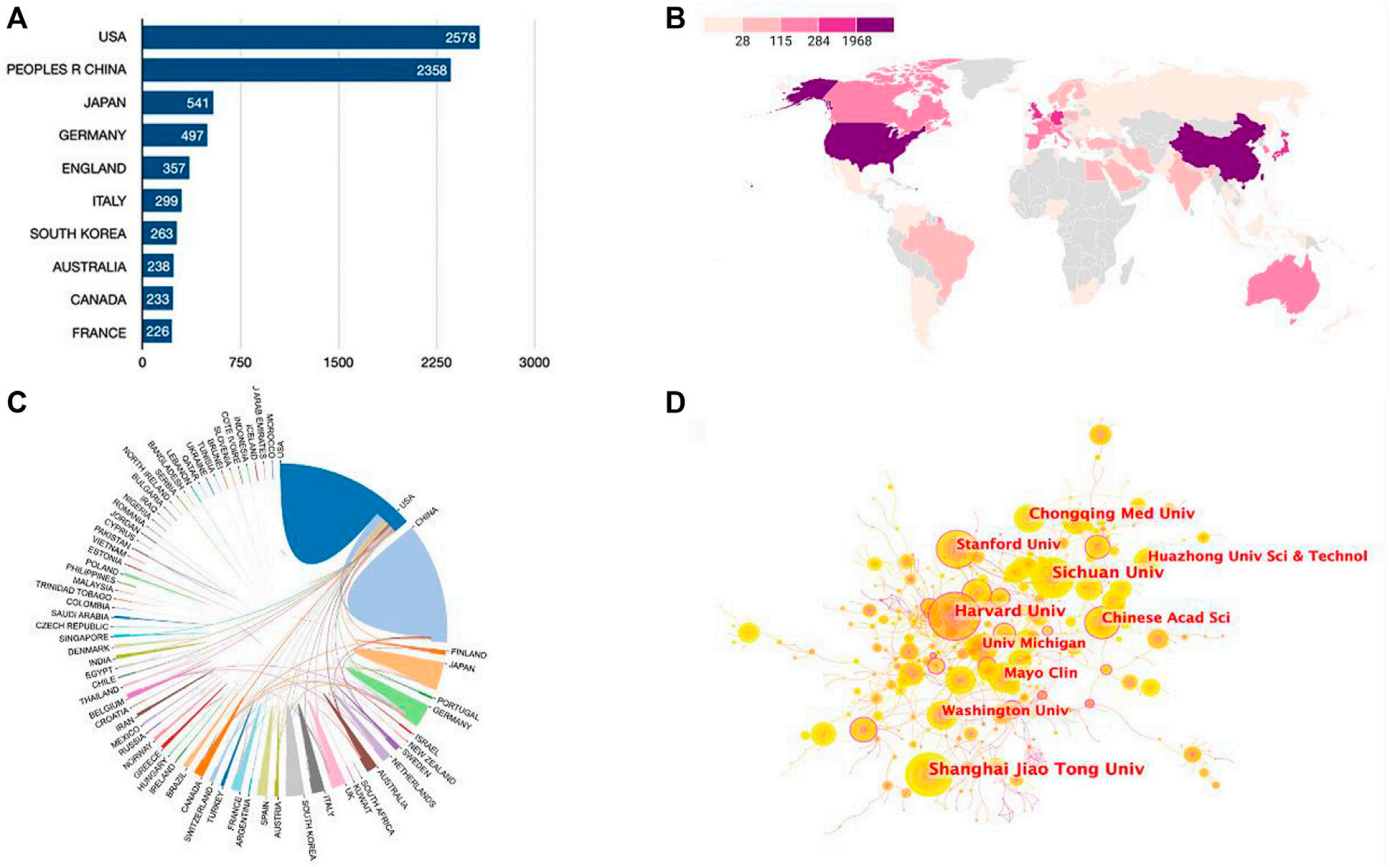
Cooperation relationships between countries/regions and institutions. **(A)** Bar graph indicating the number of Wnt in Bone-related publications in the Top10 countries/regions. **(B)** Heat map showing the distribution of Wnt in Bone-related publications in the world. **(C)** Cooperation relationships between countries/regions. **(D)** The network map of institutions involved in the Wnt in Bone research.

Publications were available in 89 countries/regions worldwide. Number of publications by year for different countries/regions is illustrated in Figure 2B (top 10 countries/regions are shown only). The United States had the greatest number of Wnt in Bone-related publications (2,578, 35.9%), followed by China (2,358, 32.8%), Japan (541, 7.5%), Germany (497, 6.9%), and England (357, 5.0%) (Figure 3A). Wnt in Bone-related publications are distributed by country in Figure 3B.

Regarding research institutions, 8 of the top 10 prolific institutions are from 2 countries: the United States and China. University of California in the United States topped the list with 223 articles and 13,128 total citations, followed by Harvard University in United States (213 articles, 21,685 total citations) and Shanghai Jiao Tong University in China (163 articles, 4,686 total citations) (Table 1). As seen in Figure 3C, there was relatively frequent collaboration between the United States and China, followed by that between the United States and Japan. Yet, the institutional network diagram indicated a low density, suggesting that these research institutions are relatively independent and need further collaboration (Figure 3D).

**TABLE 1:** The Top10 institutions contributing to Wnt and Bon research.

Institutions	Article counts	Total citations	Average citations	Country
University of California	223	13,128	58.87	United States
Harvard University	213	21,685	101.81	United States
Shanghai Jiao Tong University	163	4,686	28.75	China
SiChuan University	140	2,715	19.39	China
Harvard Medical School	138	12,304	89.16	United States
Veterans Health Administration	125	7,669	61.35	United States
National Institutes of Health	124	13,930	112.34	United States
Udice, French research universities	124	5,053	40.75	France
University of Texas System	116	10,266	88.5	United States
University of London	109	5,905	54.17	England

### 3.3 Analysis of authors and journals

A total of 28,693 authors were involved in Wnt in Bone-related publications, and the top 10 prolific authors are shown in Table 2. Wang, Yang from the Shanghai Jiao Tong University Affiliated Sixth People’s Hospital (China) ranked first (73 articles in total: 15 as first author and 4 as corresponding author). Liu, Yang from the Chinese University of Hong Kong (Hong Kong, China) ranked second (56 articles in total: 14 as first author and 2 as corresponding author).

**TABLE 2:** The Top10 most productive authors in Wnt in Bone research.

Author	Article counts	Total citations	Average citations	First author counts	First author citation counts	Average first author citation counts	Corresponding author counts	Corresponding author citation counts
Wang, Yang	73	296	4.05	15	35	2.33	4	7
Liu, Yang	56	163	2.91	14	51	3.64	2	8
Zhang, Jie	56	160	2.86	7	56	8	8	3
Li, Jie	55	393	7.15	10	172	17.2	3	2
Li, Yang	55	82	1.49	9	9	1	4	1
Zhang, Ying	54	67	1.24	7	2	0.29	6	5
Wang, Jing	49	309	6.31	9	33	3.67	5	3
Zhang, Li	48	163	3.40	4	14	3.5	2	0
Wang, Lu	44	134	3.05	10	22	2.2	6	11
Liu, Jian	43	101	2.35	5	4	0.8	6	10

A total of 261 journals appeared in the Wnt and Bone research field. Table 3 shows the characteristics of the top 10 most active journals. In the Wnt and Bone field, Bone is the most contributing journal with the most significant number of publications (217), followed by the Journal Of Bone And Mineral Research (211). Proceedings Of The National Academy Of Sciences was ranked with the highest impact factor (IF) of 12.779. Proceedings Of The National Academy Of Sciences Of The United States and Journal Of Biological Chemistry were respectively ranked first (129.81) and second (117.15) for the average number of citations, with the latter placing highest for the total number of citations. The mentioned journals are ranked Q1 in one-third of the Journal Citation Reports (JCR). Most publishers are located in the United States, followed by England.

**TABLE 3:** The Top10 most active journals that published articles in ME research.

Journal	Article counts	Country	JCR (2021)	If (2021)	Web of science category	Total cites	Average citations
Bone	217	United States	Q1	6.390	ENDOCRINOLOGY & METABOLISM	8,350	38.48
Journal Of Bone And Mineral Research	211	United States	Q2	4.626	ENDOCRINOLOGY & METABOLISM	15,251	72.28
Plos One	184	United States	Q2	3.752	MULTIDISCIPLINARY SCIENCES	6,509	35.38
Journal Of Biological Chemistry	133	United States	Q2	5.485	BIOCHEMISTRY & MOLECULAR BIOLOGY	15,581	117.15
Scientific Reports	112	England	Q2	4.997	MULTIDISCIPLINARY SCIENCES	2,546	22.73
Biochemical And Biophysical Research Communications	108	United States	Q3	3.322	BIOCHEMISTRY & MOLECULAR BIOLOGY; BIOPHYSICS	2,943	27.25
Journal Of Cellular Biochemistry	105	United States	Q2	4.481	BIOCHEMISTRY & MOLECULAR BIOLOGY; CELL BIOLOGY	5,009	47.70
Proceedings Of The National Academy Of Sciences Of The United States	81	United States	Q1	12.779	MULTIDISCIPLINARY SCIENCES	10,515	129.81
Journal Of Cellular Physiology	77	United States	Q2	6.513	CELL BIOLOGY; PHYSIOLOGY	2,754	35.77
Development	72	England	Q1	6.862	DEVELOPMENTAL BIOLOGY	7,603	105.6

### 3.4 Analysis of keyword co-occurrence

**FIGURE 4:**
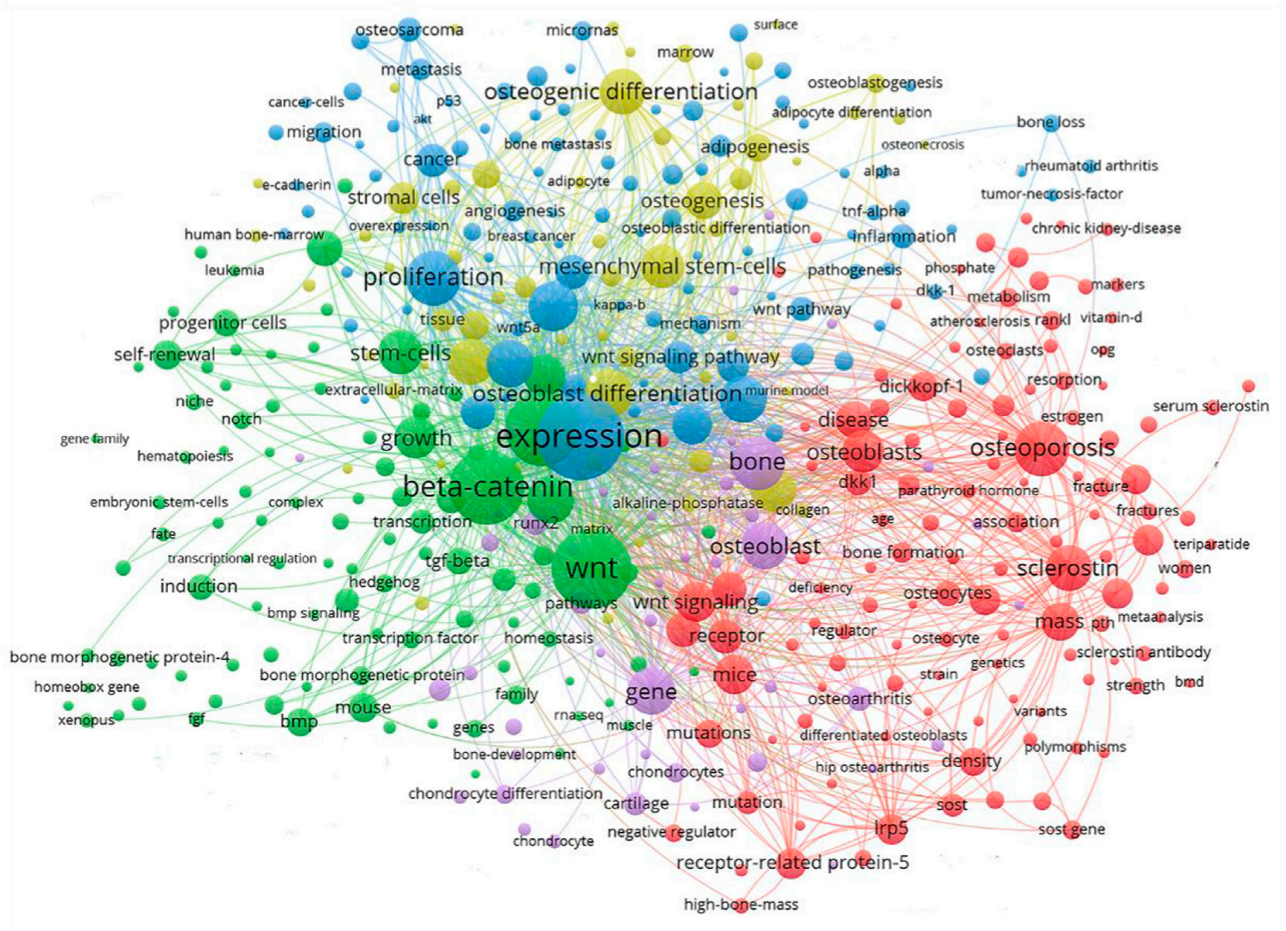
Keyword clustering map showed 901 keywords with a minimum of 10 co-occurrences and divided into 5 major clusters.

Keyword clustering analysis can be used to visualize domain-specific directions and popular subjects. A global keyword graph is created using keywords identified from the content of titles and abstracts with the VOSviewer tool. As shown in Figure 4, a total of 901 keywords with a minimum frequency of 10 were selected from 15,189 keywords, and then divided into 5 major color clusters representing the 5 research focus. The keywords in the blue cluster are: expression, proliferation, Wnt signaling pathway, cancer, osteosarcoma, metastasis, migration, inflammation, micrornas, etc. The keywords in the green cluster are: Wnt, beta-catenin, growth, self-renewal, induction, bmp, tgf-beta, mouse, progenitor cells, transcription, etc., The keywords in the red cluster are: osteoporosis, osteoblasts, disease, sclerostin, receptor, mice, dickkopf-1, mutations, mass, etc., The keywords in the purple cluster are: bone, osteoblast, gene, runx2, cartilage, protein, chondrocytes, osteoarthritis, chondrocyte differentiation, etc., The keywords in the yellow cluster are: osteogenic differentiation, mesenchymal stem-cells, osteoblast differentiation, osteogenesis, adipogenesis, stromal cells, mrrow, surface, alkaline-phosphatase, etc.

### 3.5 Analysis of cocited articles and reference cluster

**FIGURE 5:**
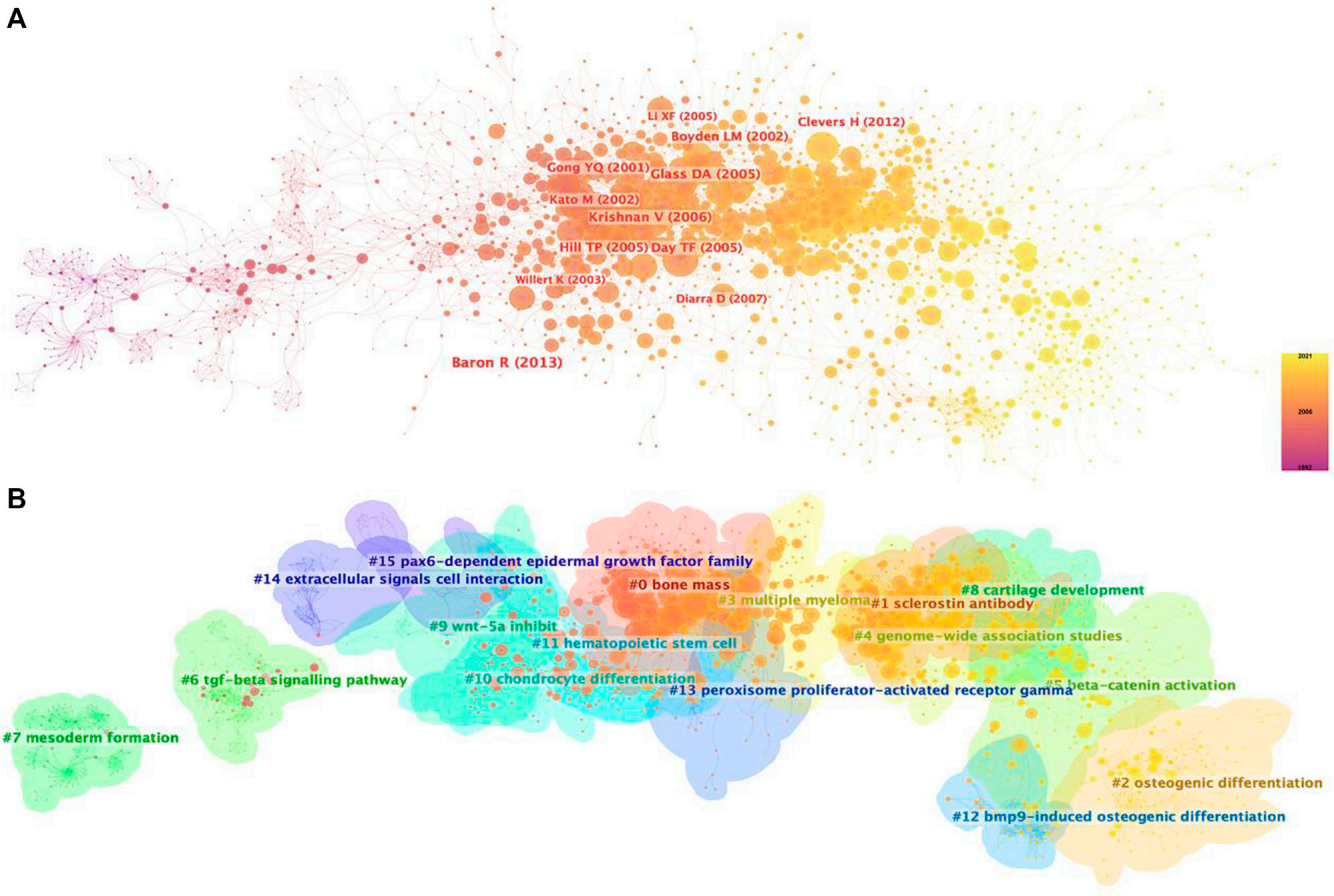
Co-citation map of Wnt in Bone. **(A)** Mapping of co-cited references on Wnt in Bone. **(B)** Clustered network map of co-cited references.

**FIGURE 6:**
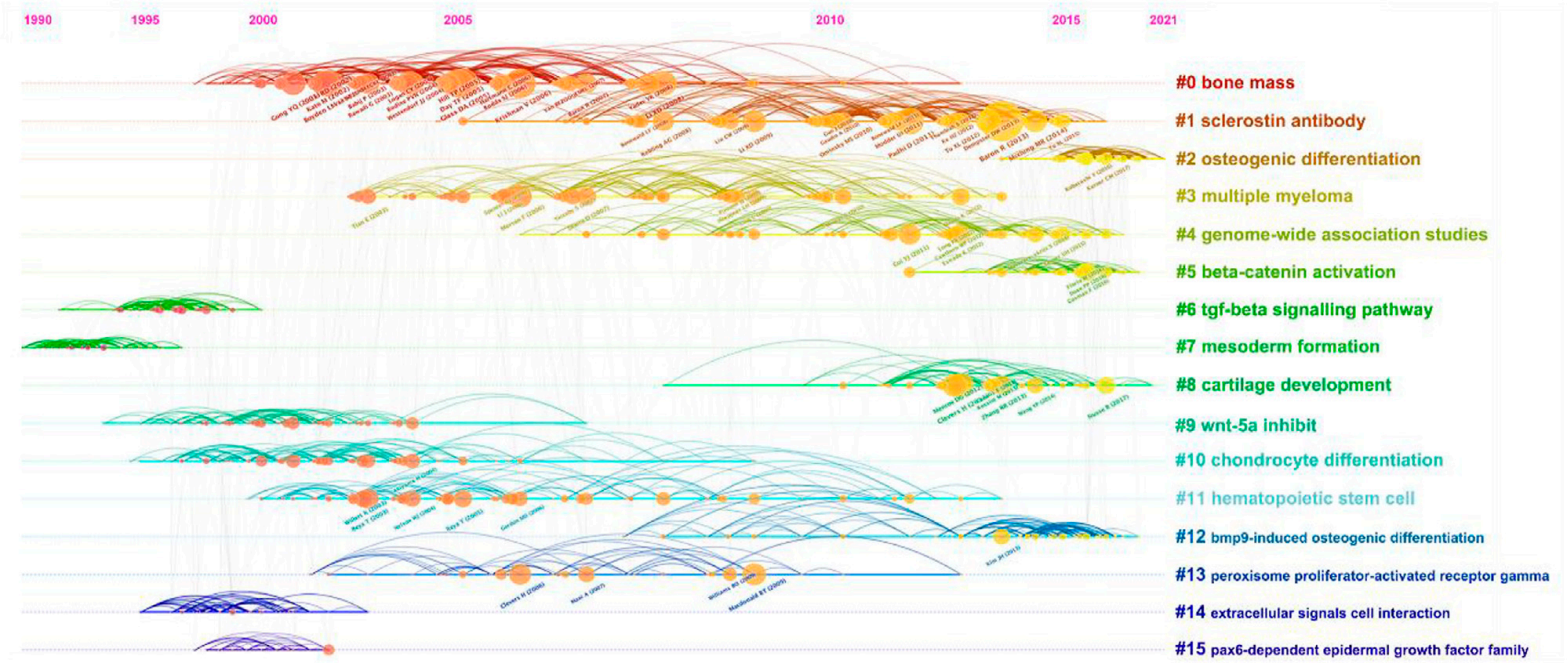
The timeline view of co-citation clusters with cluster labels.

Supported by CiteSpace software, 15 major clustering tags were formed based on cocited references, including bone mass, sclerostin antibody, osteogenic differentiation, multiple myeloma, genome-wide association studies, beta-catenin activation, tgf-beta signaling pathway, mesoderm formation, cartilage development, Wnt-5a inhibit, chondrocyte differentiation, hematopoietic stem cell, bmp9-induced osteogenic differentiation, peroxisome proliferator-actibated receptor gamma, extracellular signals cell interaction and pax6-dependent epidermal growth factor family (Figure 5B). Figure 6 shows the timeline view of these 15 cluster map, demonstrating the evolving characteristics of each cluster and supports the findings of the emerging focus in Wnt and Bone research.

### 3.6 Analysis of burst article

**FIGURE 7:**
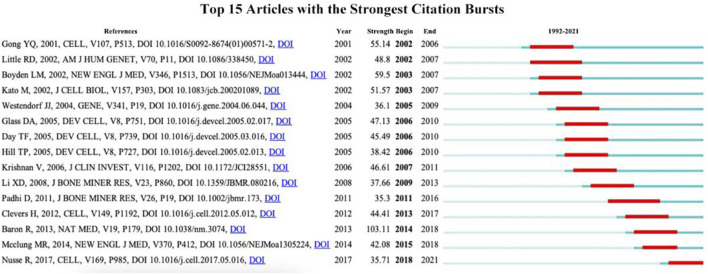
Articles with the strongest citation bursts on Wnt in Bone research from 1997–2022.

CiteSpace can be applied for detecting burst references to catch the rapid growth of frequently cited articles by scholars during a specific time period. The red line indicates the time span of the citation burst, namely, the burst period, and the blue line indicates the time interval. Articles with little or no research significance were excluded, and those representing research trends were kept. Figure 7 shows the 15 articles with the highest burst intensity for Wnt and bone-related studies between 1993 and 2022, with burst intensities ranging from 35.3 to 103.11 and burst periods of 3–5 years. Baron R’s study titled “WNT signaling in bone homeostasis and disease: from human mutations to treatments” has the highest burst strength (intensity = 103.11) with a citation burst from 2014 to 2018. Nusse R’s study published in “CELL” has the newest citation burst (intensity = 35.71) with citation bursts ranging from 2018 to 2021.

## 4 Discussion

### 4.1 Research results analysis

According to information from the WOS Core Collection, a total of 28,693 authors from 4,451 institutions in 89 countries/regions published a total of 7,184 articles related to Wnt and bone in 261 scholarly journals from 1993 to 2022. The number of publications and citations has steadily increased year by year and peaked in 2021. From 2005 to 2022, the number of publications increased more than five-fold (Figure 2), indicating growing interest in this field. While the United States is the most productive country in this field, China has equaled or even exceeded the United States in annual publications since 2015. The United States and China are the top two countries that collaborate most frequently, while the United States also actively collaborates with other countries such as Finland, Germany, and the UK. The heat map of publications (Figure 3B) suggests that more extensive cooperation and exchange between countries/regions is needed to break the imbalance of research development. Six affiliations and eight academic journals from the United States are among the top 10 affiliation structures and journals, indicating that the United States has many excellent institutions and publishers in Wnt and bone research. The top 10 journals are mainly categorized as MULTIDISCIPLINARY SCIENCES, BIOCHEMISTRY & MOLECULAR BIOLOGY, CELL BIOLOGY and ENDOCRINOLOGY & METABOLISM in WOS. The majority of manuscripts published in this field are in basic research journals, while some are in multidisciplinary or clinically relevant journals, indicating that current research is undergoing translation from basic to clinical. Half of the top 10 journals have high IF scores (>5.0), but less than half (33%) are ranked Q1 in the JCR quartile, suggesting that the quality of articles in this field needs further improvement. Researchers who wish to focus on the field of Wnt and bone can refer to the journals listed in Table 2.

### 4.2 Hotspots and frontiers

To obtain a holistic perspective of the development of the subject area, thorough analysis of keywords in specific fields and categorization are necessary (Qin et al., 2020). According to the clustering analysis (Figure 4), the Wnt pathway is found in five categories related to the bone. Co-citation reference clustering, a method in which cited references are analyzed to identify those that appear frequently, can also be employed to reveal research trends and cutting-edge developments in a given area (Figures 5, 6). Meanwhile, citation burst analysis can help trace research hotspots, core literature, and leading figures (Figure 7). Interestingly, several authors in the citation burst analysis also appear in the Mapping of co-cited references, including Gong YQ, Boyden LM. Kato M, Day TF, Hill TP, Krishnan V, Clevers H and Baron R (Figures 5A). Through analysis of keywords, citations, and the top ten most cited literature, this paper summarizes the current hotspots and prospects for research focused on skeletons within the Wnt pathway.

#### 4.2.1 Bone mass regulation by Wnt

Studies on changes in bone volume are the most researched aspect of Wnt-related bone studies, as shown in Figure 6. Among the 15 articles with the highest citation burst intensity, ten were related to bone mass (Figure 7), and four of the top ten frequently cited references were also linked to bone mass (Table 4). The Wnt signaling pathway includes low-density lipoprotein receptor-related protein 5 (LRP5), one of the receptors. LRP5 is considered to be a vital molecule in Wnt signaling as it interacts with Wnt ligands, along with its homologous family member LRP6 and other proteins, in receptor-mediated endocytosis of lipoprotein and protein ligands (Joiner et al., 2013). It regulates various developmental processes in embryogenesis and maintains physiological homeostasis during maturation (Audrey Koay and Brown, 2005). Loss-of-function mutations in LRP5 were found by Gong et al. to cause an autosomal recessive disorder called osteoporosis-pseudoglioma syndrome (OPPG) 20 years ago, which is characterized by low bone mass and skeletal fragility (Gong et al., 2001). On the other hand, acquired mutation in LRP5 function leads to high bone mass trait (HBM) in families, which is associated with the loss of DKK1 antagonism after an amino acid substitution in LRP5’s first β-propeller structural domain (LRP5_G171V_) (Boyden et al., 2002; Little et al., 2002). In humans, inherited bone mass changes and skeletal diseases such as osteoporosis were demonstrated to be caused by various variants in the LRP5 gene (Van Wesenbeeck et al., 2003). Animal models determined the mechanism of action of LRP5 mutations in the osteoblast spectrum (Kato et al., 2002; Babij et al., 2003; Yadav et al., 2008), generating LRP5^−/−^ mice that exhibit early-onset osteoporosis and sustained embryonic ocular angiogenesis, the former associated with reduced bone formation and mineralization due to reduced osteoblast proliferation in postnatal mice (Kato et al., 2002). Furthermore, mice with the LRP5_G171V_ mutation exhibit higher bone mass and bone strength than wild-type mice, which is associated with enhanced bone anabolism mediated by increased osteoblast activity and survival (Gong et al., 2001; Babij et al., 2003). Taken together, the specific mechanism by which LRP5, an important molecule in the Wnt signaling pathway, regulates bone mass by disrupting endogenous LRP antagonists such as DKK1 or sclerostin leads to increased Wnts signaling activity and the ability to stimulate osteogenesis (Balemans et al., 2007; van Bezooijen et al., 2007; Li et al., 2008), further solidifying the central role of LRP5 in Wnt pathway-mediated bone metabolism.

**TABLE 4:** The Top10 most cited articles in Wnt and Bone research.

Title	Authors	Year	Journal	Total citations
LDL receptor-related protein 5 (LRP5) affects bone accrual and eye development	Gong,YQ	2001	Cell	1,672
Wnt proteins are lipid-modified and can act as stem cell growth factors	Massague, Joan	2003	Nature	1,666
Geometric cues for directing the differentiation of mesenchymal stem cells	Kilian, KA	2010	Proceedings Of The National Academy Of Sciences Of The United States	1,322
Wnt/beta-catenin signaling in mesenchymal progenitors controls osteoblast and chondrocyte differentiation during vertebrate skeletogenesis	Day, TF	2005	Developmental Cell	1,260
High bone density due to a mutation in LDL-receptor-related protein 5	Boyden, LM	2002	New England Journal Of Medicine	1,201
Canonical Wnt signaling in differentiated osteoblasts controls osteoclast differentiation	Glass, DA	2005	Developmental Cell	1,157
The role of the Wnt-signaling antagonist DKK1 in the development of osteolytic lesions in multiple myeloma	Tian, E	2003	New England Journal Of Medicine	1,106
Sclerostin binds to LRP5/6 and antagonizes canonical Wnt signaling	Li, XF	2005	Journal Of Biological Chemistry	1,006
Dickkopf-1 is a master regulator of joint remodeling	Diarra, Danielle	2007	Nature Medicine	982
Comparative characteristics of mesenchymal stem cells from human bone marrow, adipose tissue, and umbilical cord blood	Wagner, W	2005	Experimental Hematology	938

Several potential targets for regulating bone mass through the Wnt pathway have been identified, such as antibodies to sclerostin and/or DKK1 (Li et al., 2011), NELL1 (James et al., 2015), Lithium (Amirhosseini et al., 2018), and Sirtuins (Zainabadi et al., 2017). These targets act on different components of the pathway, such as the LRP5/6 co-receptor, β1 integrins, and GSK3β enzyme (Appelman-Dijkstra and Papapoulos, 2018). Only sclerostin antibodies have advanced to clinical trials, while other Wnt antagonists are still in preclinical development due to their nonspecific effects on skeletal tissues. Sclerostin, a glycoprotein mainly encoded by the SOST gene and secreted by osteoblasts, binds to LRP5/6 co-receptor and inhibits Wnt/β-catenin pathway with DKK1, affecting bone formation and remodeling. Several clinical trials have tested Sclerostin antibodies, such as Blosozumab (McColm et al., 2014), BPS-804 (Glorieux et al., 2017), SHR-1222 (Gao et al., 2021) and Romosozumab (AMG 785) (Yu S. et al., 2022). Romosozumab (AMG 785), which stimulates both bone formation and resorption, has shown superior efficacy for severe osteoporosis in postmenopausal women (Balemans et al., 2007; Padhi et al., 2011). A phase III clinical trial (the FRAME study) showed that romosozumab reduced the risk of vertebral fracture by 73% (*p* < 0.001) and clinical fracture by 36% (*p* = 0.008) at 12 months compared to placebo. This effect persisted at 24 months (*p* < 0.001), even after switching to denosumab in both groups, with a 75% lower risk of vertebral fracture in the romosozumab group. Bone turnover markers confirmed that romosozumab increased bone formation and decreased bone resorption (Cosman et al., 2016). Another trial (the ARCH study) found that romosozumab combined with alendronate lowered the risk of new vertebral fracture by 48% (*p* < 0.001) after 24 months of separate treatments than alendronate alone, and also reduced the risk of other fractures including clinical fracture (27%), vertebral fracture (19%), and hip fracture (38%) (Saag et al., 2017). Therefore, sclerostin inhibitors have a rapid and powerful effect on bone mass, and romosozumab was approved by the FDA and EMA in 2019 for treating osteoporosis in postmenopausal women at high risk of fracture. However, the cardiovascular event imbalance observed in the romosozumab group in the ARCH study is a safety concern that needs urgent confirmation and resolution (Saag et al., 2017). Yu Y et al. (Yu Y. et al., 2022) suggested that inhibiting sclerostin ring 3 expression could increase bone formation without cardiovascular risk. Moreover, long-term pharmacodynamic studies of romosozumab indicated that its bone formation effect diminished over time, possibly due to another WNT inhibitor (Florio et al., 2016). Thus, the efficacy of neutralizing antibodies to DKK1 synergizing Romosozumab was confirmed in rodents and non-human primates (Florio et al., 2016). In conclusion, promising therapies for osteoporosis targeting the Wnt pathway may involve bispecific antibody inhibitors of DKK1 and sclerostin loop 3.

#### 4.2.2 Regulation of Wnt in multiple myeloma

Wnt signaling in multiple myeloma (MM) is also of interest to scholars. In the WoSCC core database, there were 243 publications related to Wnt and MM, and two of the top 10 co-cited publications were related to MM. MM is a malignant plasma cell disease that is characterized by the production of monoclonal immunoglobulins. It often leads to osteolytic bone disease, causing bone pain and pathological fractures (van Andel et al., 2019). The normal human skeleton is mainly regulated by sclerostin secreted by osteocytes and wnt antagonists such as DKK1, which modulate osteoblast-mediated new bone formation (Robling and Bonewald, 2020). In MM patients, MM-associated bone disease (MBD), characterized by osteolytic lesions, is mainly triggered by the high upregulation of DKK1 secreted by malignant plasma cells in the bone marrow microenvironment (Zhou et al., 2013). DKK1 inhibits canonical Wnt signaling of osteoblasts, leading to reduced osteoblast differentiation, upregulation of the OPG: RANKL ratio, and an increase in the activity and number of osteoclasts. This results in insufficient bone formation compensation and ultimately produces osteolytic lesions (van Andel et al., 2019; Jaschke et al., 2020). This key mechanism is illustrated by Tian et al., a highly cited study, who found that elevated serum levels of DKK1 were strongly associated with the presence of bone disease in MM (Tian et al., 2003). Although the reason for the high circulating levels of DKK1 seen in MM patients is not yet fully explained, a monoclonal antibody targeting DKK1, BHQ880, has been developed in view of its key role in the pathology (Iyer et al., 2014). The safety and efficacy of BHQ880 was also evaluated in a phase I clinical trial conducted by Iyer et al., who observed that BHQ880 was well tolerated and showed a general trend of increasing BMD over time (Iyer et al., 2014). In conclusion, anti-DKK1 therapy has great clinical potential as a novel therapy for mm, with further large sample size randomized controlled trials of BHQ880 and other targets of the Wnt pathway [e.g., R-spondin (Spaan et al., 2018), ROR2 (Frenquelli et al., 2020) and CK1α/RUNX2 (Fregnani et al., 2022)] emerging.

#### 4.2.3 Bone and joint diseases in Wnt

In addition to differentiating into osteoblasts, mesenchymal stem cells (MSCs) also differentiate into chondrocytes, adipocytes, myogenic cells, and fibroblasts. This process is regulated by tissue-specific transcription factors (Huang et al., 2022). Among these, the development and maturation of cartilage are closely associated with various bone and joint diseases, which has naturally attracted the attention of researchers. However, the role of the Wnt signaling pathway in regulating cartilage homeostasis is complex. Both overexpression and loss of Wnt/β-linked proteins in articular cartilage can lead to joint damage (Lories et al., 2013). For instance, the activation of typical or atypical Wnt ligands (Wnt5a, Wnt8a, etc.,) promotes cartilage catabolism (van den Bosch et al., 2015; Huang et al., 2017). In contrast, Wnt16 maintains a balanced canonical WNT signaling and prevents the development of osteoarthritis (OA) (Nalesso et al., 2017). Additionally, the knockdown of Wnt inhibitors, such as secretory frizzled-related protein-3 (sFRP-3), is associated with increased susceptibility to OA (Loughlin et al., 2004; Lories et al., 2007), and genetic variants of DKK1 are significantly associated with joint destruction in patients with rheumatoid arthritis (RA), who also have higher levels of functional serum DKK1 (Diederik et al., 2013). Conversely, serum-mediated suppression of Wnt signaling is reduced in patients with ankylosing spondylitis (AS), and functional DKK1 levels are strongly associated with the mechanism of ligamentous tubercle formation in AS. DKK1 has potential as a biomarker for spondyloarthritis (SpA) (Daoussis et al., 2010; Gisela Ruiz et al., 2012). As for targeting the Wnt signaling pathway to improve the treatment of arthritis, it is challenging (Stampella et al., 2017). However, a recent 52-week phase IIa clinical trial demonstrated that the Wnt signaling pathway modulator lorecivivint (SM04690) has good efficacy and safety in the treatment of OA (Yazici et al., 2020). This should encourage the development of other emerging therapeutic approaches targeting the Wnt signaling pathway (Wang et al., 2019).

## 5 Limitations

However, there are some limitations to this study. Firstly, the data were only from the Web of Science Core Collection (WoSCC) database, and the lack of other databases or literature published in non-SCI journals could slightly affect the results of the study. Secondly, the literature analysis using visualization tools only considered the quantity and citations of the literature and did not fully consider factors such as the quality and academic value of the literature. Thirdly, the three analysis tools used in this study, namely, CiteSpace, VOSviewer, and bibliometrix, cannot entirely replace systematic searching. Despite these limitations, this literature analysis is unlikely to show much publication bias and provides a comprehensive overview of Wnt-related bone research.

## 6 Conclusion

This literature analysis presents the first scientific and systematic review of 7,184 publications on Wnt-related bone research during 1992–2022 and analyzes literature information from different countries, institutions, authors, keywords, and journals to reveal the development and future research trends in the field. Publications in Wnt-related bone research have accumulated rapidly over the past 30 years and have remained stable in recent years. This report finds that the research hotspots in this field are mainly focused on the mechanism study of bone mass regulation by Wnt pathway-related molecules, the feasibility study of sclerostin antibody against osteoporosis and the mechanism exploration of Wnt pathway and joint diseases. Future research trends in this area include safety and targeting studies on sclerostin antibodies, potential target drugs for multiple myeloma (MM), and high-quality clinical trials for the treatment of osteoarthritis (OA). In summary, researchers in this topic will delve into the mechanisms of Wnt and skeletal-related diseases, with the potential for further translation to clinical research.

## Data Availability

Publicly available datasets were analyzed in this study. This data can be found at Web of Science (WOS) core database by our search formula TS = (Wnt (Topic) or Wnt signaling pathway (Topic)) and (bone (Topic)): https://webofscience.clarivate.cn/wos/woscc/summary/b1d33977-2d62-4deb-a734-d6f3e4a02fb8-b3e36e4f/relevance/1, and the literature analyzed is available in the Supplementary Materials.
